# Preliminary Experiments on Human Sensitivity to Rhythmic Structure in a Grammar with Recursive Self-Similarity

**DOI:** 10.3389/fnins.2016.00281

**Published:** 2016-06-28

**Authors:** Andreea Geambaşu, Andrea Ravignani, Clara C. Levelt

**Affiliations:** ^1^Leiden University Centre for Linguistics, Leiden UniversityLeiden, Netherlands; ^2^Leiden Institute for Brain and Cognition, Leiden UniversityLeiden, Netherlands; ^3^Artificial Intelligence Lab, Vrije Universiteit BrusselBrussels, Belgium; ^4^Department of Cognitive Biology, Faculty of Life Sciences, University of ViennaVienna, Austria

**Keywords:** statistical learning, rhythm, recursion, artificial grammar learning, rhythm perception, Lindenmayer system, L-system, Fibonacci grammar

## Overview

We present the first rhythm detection experiment using a Lindenmayer grammar, a self-similar recursive grammar shown previously to be learnable by adults using speech stimuli. Results show that learners were unable to correctly accept or reject grammatical and ungrammatical strings at the group level, although five (of 40) participants were able to do so with detailed instructions before the exposure phase.

## Introduction

Processing of hierarchical structures has been proposed as a uniquely human ability, a hallmark of the linguistic system that distinguishes human language from animal communication systems (Hauser et al., 2002; Martins, 2012). Recursion is often considered the pinnacle of human-specific hierarchical structures (Hauser et al., 2002). Artificial Grammar Learning experiments have shown that adult participants are able to learn the context-free grammar A^n^B^n^, whose generation requires hierarchical rules, even without the need for semantic information (Lai and Poletiek, 2013). Parsing and generalizing grammars like A^n^B^n^ requires detection that a structure, e.g., AB, is embedded between elements of another structure, e.g., A…B. Other species have not been shown unequivocally to be able to learn on the basis of the center-embedding principle required of A^n^B^n^ (rather than using other strategies, Corballis, 2007; van Heijningen et al., 2009; Beckers et al., 2012; Poletiek et al., 2015; Ravignani et al., 2015), which is taken as evidence that processing of recursion is a human-specific capacity.

**Figure 1:**
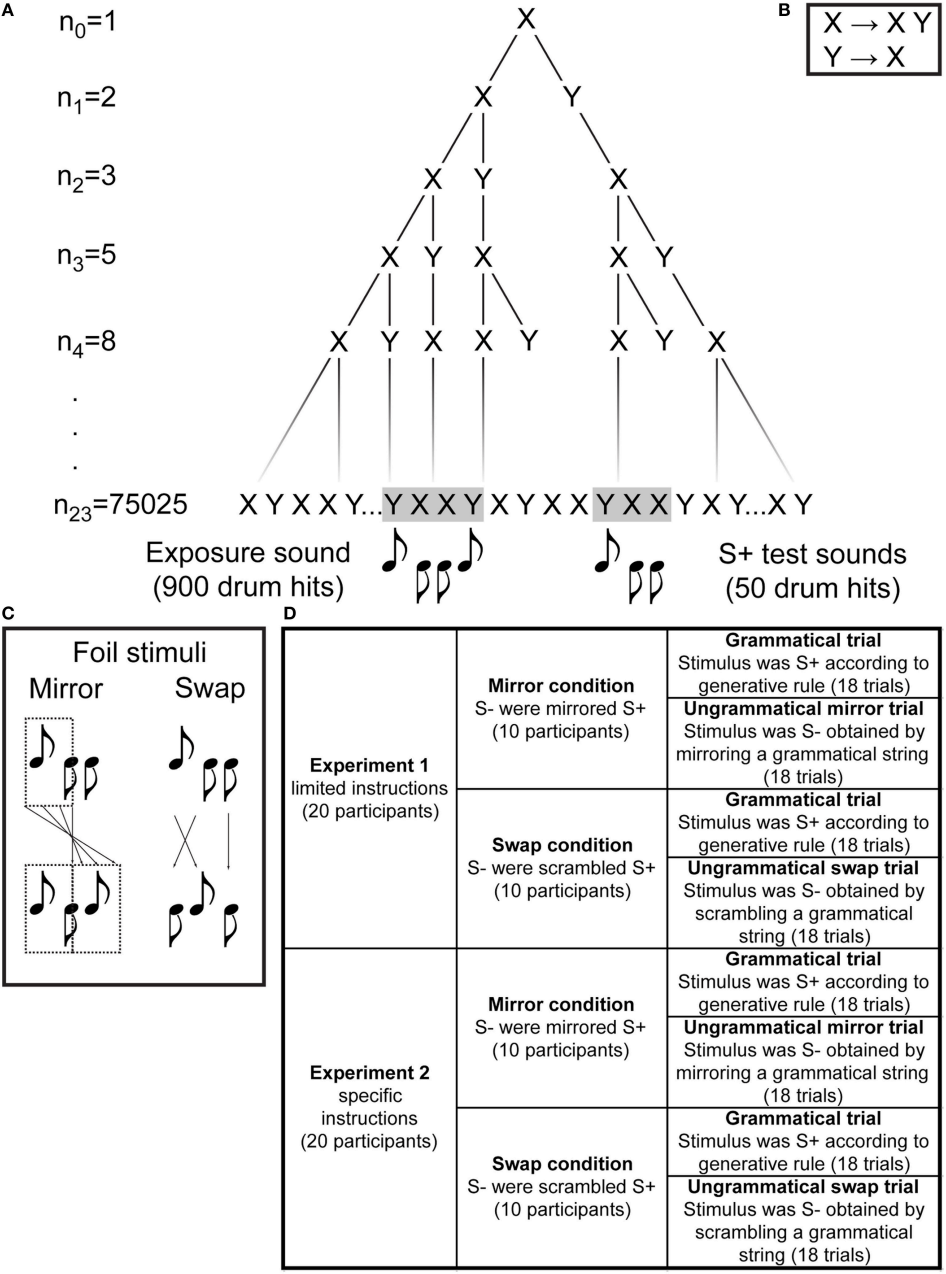
**A derivation of the target Fibonacci grammar at the first four iterations and at the final 23rd iteration used to generate the exposure and test stimuli (A), the rewrite rules of the grammar (B), the makeup of the two foil grammars (C), and an overview of the two experiments reported with their two respective foil test conditions (D)**. We use upward and downward note stems to differentiate between the two drum sounds.

Yet to what extent learning of an A^n^B^n^ grammar can be taken as evidence for processing recursive information at all is debated. Some researchers argue that human participants could in fact use simpler strategies, such as counting and matching the number of As and Bs in a test sequence (Hochmann et al., 2008; Zimmerer et al., 2011), while others argue that despite different strategies, the same core operations are nonetheless necessary (Fitch and Friederici, 2012; Fitch, 2014). Saddy (2009) proposed that a more suitable grammar for the investigation of recursive processing may be Lindenmayer grammars, or L-systems. Uriagereka et al. (2013) have proposed that these grammars are suitable for between-species comparative work because they generate utterances that can be infinitely long and produce a “rhythm” when recognized. L-systems were first proposed by Lindenmayer to describe algae cell growth (Lindenmayer, 1968; Lindenmayer and Rozenberg, 1972) and have since been used to describe and recognize different plant structures (Samal et al., 1994). L-systems have rewrite rules that occur in parallel and have no terminal symbol, indicating that they can produce infinite sequences (Figure 1A). Because of their hierarchical structure and recursive properties, they are an interesting grammar to use in testing recursive processing. In her dissertation, Shirley (2014) began to explore the learnability of Fibonacci grammars, a subgroup of L-systems, that at each iteration produce sequences with lengths corresponding to Fibonacci numbers. She found that after a 3-min training with a Fibonacci grammar composed of syllables *bi* and *ba*, participants were able to correctly accept grammatical 10-s-long structures, and correctly reject ungrammatical ones. However, how participants processed the stimuli in Shirley's task is not clear yet. A possible rhythm-based strategy may have been used by participants to recognize a pattern in sounds generated by recursive branching, using rhythmic structure, i.e., how durational events are grouped and perceived hierarchically based on their relative accentuation. When presented with sequences of acoustic events occurring at constant time intervals (i.e., isochronous, as in Shirley, 2014), humans tend to group these events. Grouping often occurs when events are differentially accented, that is, marked by differing pitch or intensity (e.g., strong-weak-weak, Hay and Diehl, 2007).

The detection of a specific rhythmic pattern might be the mechanism participants draw upon to detect recursive structures such as those tested here. Syllables in Shirley (2014) differed by their vowel quality, with possibly some non-systematic variation in fundamental frequency and intensity. If detection strategies based on rhythmic features were used to learn Shirley's grammars, participant tested with percussion sounds (enhancing the recursive rhythmical structure of the stimuli) instead of speech syllables should show similarly high or even better performance, as the non-temporal rhythmic cues (intensity or pitch accentuation) would be enhanced, while violations in interstimulus intervals would disrupt the rhythmic detection strategy and hence grammar recognition (Shirley, 2014).

Can a complex pattern, recursively and hierarchically organized according to an L-system, be learned on the basis of a rhythmical strategy? We tested this hypothesis by enhancing the rhythmic quality of the sequences by using drum sounds differing in pitch and intensity, instead of syllables. This work thus constitutes the first study on rhythm perception using L-systems^1^. We conducted two experiments (Figure 1D), each with two conditions (two types of foil grammars) to evaluate the learnability of the L-system grammars. Between our two experiments, we also varied instructions, to further explore whether the method of presenting the exposure stimuli had an effect on learning ability. Based on previous work by Saddy (2009) and Shirley (2014) we expected that participants would pick up on the rhythmic nature of the structures, and be able to discriminate grammatical from ungrammatical strings. Our results indicate that for the majority of our participants, rhythm alone may not be enough to learn this type of grammar; musical background, age, instruction, and the specific types of foil grammars may all be contributing factors.

## Methods and materials

Two experiments were conducted, using Fibonacci grammars similar to those used in Saddy (2009) and Shirley (2014). The experiments consisted of an exposure phase and a test phase. During the exposure phase, participants passively listened to a sequence of kick and snare drum sounds following a Fibonacci grammar. During the subsequent test phase, participants were asked to indicate whether the test item (composed of the same kick and snare sounds) corresponded to the grammar from the listening phase, and to rate their certainty. The two experiments (Experiments 1 and 2) differed only in the detail of instruction given to participants. Instructions in Experiment 2 were more detailed than those in Experiment 1 (see Procedure). Each of the experiments consisted of two conditions (Mirror and Swap), in which each of the ungrammatical test items differed from the target Fibonacci grammar in different ways (see Stimuli).

### Participants

Forty students (nine males; age range 18–32, *M* = 22, *SD* = 3.05) from Leiden University participated, *N* = 20 in Experiment 1 and *N* = 20 in Experiment 2. Participants were recruited via the SONA participant recruitment website of Leiden University. None of the participants had hearing problems or were dyslexic. Participants had various linguistic backgrounds, with all participants speaking at least one foreign language. They also had varying degrees of musical experience. The study was approved by the Ethical Committee of the Faculty of Social Sciences at Leiden University. Participants signed an informed consent form before taking part and were fully debriefed on the intention of the study upon completion of the experiment. They received course credits or monetary compensation for participating.

### Stimuli

The Fibonacci sequences were made of simple drum sounds: a kick (average intensity 78 dB; sound X) and a snare (average intensity 66 dB, sound Y), each 200 ms in duration. See Figure 1B for the Fibonacci grammar's rewrite rules.

An exposure string was created using a series of custom-written Python scripts which created a large iteration of the Fibonacci grammar (*n* = 23, resulting in a 75025-element-long string). From this initial sequence, a 900-element (3-min-long) sequence was extracted and used for the habituation phase. Grammatical test items (50 elements, 10 s long) were extracted from the remaining sequence such that each grammatical string was unique.

Two modifications of the Fibonacci sequences were used as foil grammars. The first will be referred to as a Swap sequence. A Swap sequence consisted of a sequence taken from the remainder of the initial 75025-long sequence, in which a randomly-selected X and an adjacent Y from the string were switched, subject to the constraints that the swap would (i) produce a different string and (ii) not introduce an easily-detectable YY bigram (Figure 1C). For example, if the Fibonacci iteration *n*_3_ is X**YX**XY (see Figure 1A), its corresponding Swap sequence may be X**XY**XY. The second foil sequence will be referred to as a Mirror sequence. A Mirror sequence consisted of the Fibonacci sequence that was cut in half; this first half of the sequence was mirrored and replaced the original second half (Figure 1C). For example, if the Fibonacci iteration *n*_5_ is XYXXYX**Y**XXYXXY (Figure 1A), its corresponding Mirror sequence would be: XYXXYX**Y**XYXXYX, where the seventh element (Y, bold) is treated as the point of mirroring. In order to avoid introducing more than two repetitions of the X element, or more than one repetition of the Y element, the point of mirroring varied by sequence, and thus mirror sequences could be either 50 or 51 elements long.

The composition of the foil grammars ensured that they never occurred in the habituation sequence, nor could they have ever occurred in any shorter iterations of the Fibonacci grammar. They also ensured that the grammatical and ungrammatical items were as similar as possible with respect to their local (element adjacency) and global (distribution of Xs and Ys) properties, thus preventing participants from solving the task by using simpler methods such as counting.

### Materials

The experiments were conducted on a computer running Windows 7, with a 17-inch monitor (refresh rate: 60 Hz; resolution: 1280 × 1024 pixels). Participants sat ~50 cm from the screen in a quiet room and listened to the stimuli via headphones (Sennheiser HD 201). The experiment was programmed and run in Praat (Boersma and Weenink, 2014) and participant responses were registered via mouse clicks.

### Procedure

In Experiment 1, participants were first presented with the following instruction: “You will now hear a 3-min-long rhythmic sequence. Listen carefully. When the sounds stop, press the spacebar to proceed to the test phase.” Participants in Experiment 2 were presented with more specific instructions: “You will now hear a 3-min long rhythmic pattern. Listen carefully. You will have to distinguish between this pattern and another pattern in the test phase. When the sounds stop, press the spacebar to proceed to the test phase.”

Within each experiment (Figure 1D), an equal number of participants was randomly assigned to the Mirror condition or the Swap condition (*n* = 10 per condition per experiment). In both conditions, the participants listened to the same L-system exposure sequence for 3 min. During the exposure phase the display was gray and showed a black fixation cross. After the exposure phase, the testing phase began. Participants were then presented with the following instructions: “The test phase will now begin. You will hear 36 test sounds. For every sound, listen carefully and indicate whether it follows the same rhythm as during the listening phase. Rate your certainty on a scale of 1 to 5. 1 = definitely no; 2 = probably no; 3 = not sure; 4 = probably yes; 5 = definitely yes. Only answer when the sound has finished playing.” During the test phase, participants in both the Mirror and the Swap condition were tested on their ability to discriminate between 10-s-long grammatical L-system sequences and ungrammatical sequences (Mirror or Swap sequences, depending on condition). In both conditions, they were instructed to indicate whether the sequences they heard followed the same rhythm as the sequences they had heard during the listening phase. The instructions appearing on the screen during playback of each test item were as follows: “Does this sound follow the same rhythm as in the listening phase? How sure are you?.” Participants could then answer by clicking on one of two boxes with the words YES or NO. For their sureness response, they clicked on one of five boxes with numerals 1 (definitely no) through 5 (definitely yes).

Upon completion of the experiment, participants filled in a questionnaire, which inquired about their sex, age, hearing, dyslexia, languages spoken, handedness, musical training, and education level and background. They were subsequently debriefed on the purpose of the study and any questions they had were answered.

## Descriptive statistics and results

There are two types of correct answers, namely a correct acceptance of a grammatical L-system sequence, and a correct rejection of an ungrammatical foil sequence. Thus, we analyzed correct responses both overall and comparing acceptances and rejections.

At the group level, when pooling across participants, the number of correct responses was at chance for each of the four groups (1 sample *t*-test, all *t* < 1.8, all *p* > 0.12). For each experiment and in each condition, performance did not differ between correct acceptances of grammatical and correct rejections of ungrammatical stimuli (paired samples *t*-test, all |*t*| < 1.7, all *p* > 0.13); also reaction times did not differ (all *t* < 0.71, all *p* > 0.49).

**Figure 2:**
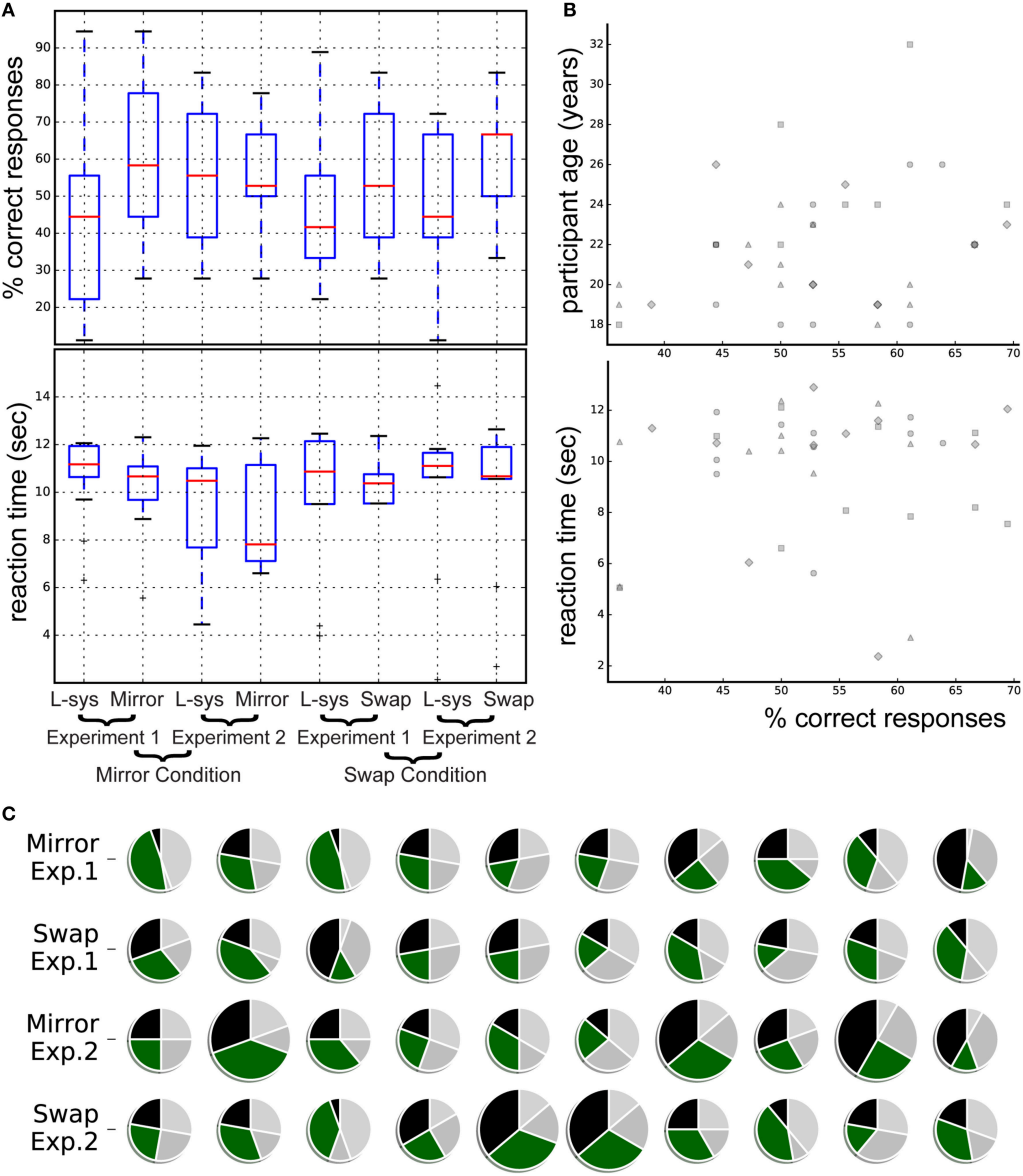
**Summary of participants' performance at group (A) and individual (B,C) level**. **(A)** Boxplot of percentage correct responses by experimental condition (Mirror vs. Swap), experiment number (limited vs. detailed instructions), and stimulus type (L-sys denotes a correct acceptance of a grammatical stimulus and Swap or Mirror denotes a correct rejection of an ungrammatical stimulus). **(B)** Individual % of correct responses is plotted against participant age and reaction time. Marker shapes denote experimental groups and conditions: mirror group without (circle) and with (square) specific instructions; swap group without (triangle) and with (diamond) specific instructions. **(C)** For each experiment, condition and participant, correct (black and green) and incorrect (silver and light gray) acceptances/rejection of grammatical and ungrammatical stimuli. Larger pies denote the five participants showing significance at an individual level.

For each of the four groups (see Figure 2), we did a Spearman correlation (uncorrected) between % correct responses and:

- Median reaction time (correlations between −0.03 and 0.12, all *p* > 0.72)
- Age of participant (correlations between −0.18 and 0.38, all *p* > 0.27)
- Certainty of response (correlations between −0.16 and 0.43, all *p* > 0.21)
- Musical training (correlations between −0.11 and 0.39, all *p* > 0.26)
- Sex (correlations between −0.41 and 0.56, all *p* > 0.08).

Analyses of individual performances showed that five participants correctly classified stimuli above chance. A Fisher exact test revealed that each of these five participants significantly more often than chance associated correct Fibonacci-grammatical stimuli as similar to the sequences heard in the exposure phase and foils as dissimilar to the sequences heard during the exposure phase (one-sided, all *p* < 0.05, all prior odds ratio using Maximum Likelihood Estimate > 4.0). These were participants numbers 30 and 31 (Experiment 2, Swap group) and numbers 22, 33, and 37 (Experiment 2, Mirror group). Interestingly, all these participants received detailed instructions. Moreover four out of five reported having musical training (12 out of our 40 participants reported musical training).

## Discussion and future experiments

Our experiments did not show that, at the group level, participants were able to learn the Fibonacci grammars and discriminate them from either the Mirror or Swap foil grammars. At the individual level however, there were five participants in Experiment 2 who correctly identified grammatical and ungrammatical strings above chance level, suggesting that with specific instructions participants may be able to discriminate the grammatical from ungrammatical strings. Of those who did perform above chance level, most had received musical training, adding weight to the argument that rhythm perception may be involved in learning this type of grammar. However, the question remains as to why most of our participants were not able to discriminate grammatical and ungrammatical strings, while the participants in Shirley (2014) were able to do this.

The very limited proficiency our participants achieved may be due to the fact that the foil grammars were too similar to the target grammar to be discriminated. While our exposure grammars were similar to those used in Saddy (2009) and Shirley (2014), our foil grammars differed in that ours did not include repetitions of both Xs and Ys, and thus could not be discriminated using repetition detection. By making the difference between target and foil grammar more subtle to avoid this method of discrimination, it might be that some of our foils were substrings of the Fibonacci-grammatical space, generated by one of the infinite iterations of the rewrite rules (Krivochen and Saddy, personal communication). This would have made discrimination between the target and foils more difficult in our experiment than in the experiments by Saddy (2009) and Shirley (2014), in which foils were part of the L-system space but not Fibonacci-grammatical. We can therefore not conclude whether or not participants are able to learn a Fibonacci grammar when presented with musical sounds. In future research, in order to be able to draw conclusions about whether musical rhythm differs from linguistic rhythm, and whether participants are able to use some sort of rhythmic structure to learn Fibonacci grammars (rather than surface properties of the stimuli) foil grammars should be calibrated to an optimal tradeoff between the structural properties of Shirley's foils and the surface properties of those used here. In addition, a different paradigm, such as Serial Reaction Time or EEG, may help illuminate what cues in the sequence participants attended to and at which point they detect an error.

In addition, several important points for consideration in future experiments are raised by our results. First, the individuals who performed above chance in correctly identifying grammatical and ungrammatical sequences, all took part in Experiment 2, where instructions were more specific than in Experiment 1. Instructions in Experiment 2 were also more in line with Shirley's instructions, letting participants know before training that they would later have to judge the correspondence between the test items and the exposure sounds. Our instructions did, however, differ from Shirley's in that Shirley used the word “language rule” whereas in our experiments, the term “rhythmic pattern” was used in order to potentially push participants even further in focusing on the rhythm of the sequences. The different terms may prime participants to listen to and learn about the same exposure grammars in different ways. Future experiments should thus take instruction into account as a factor. Furthermore, another factor that should be taken into account and balanced in the future is age of participants; although not significant in the statistical analysis, older participants may perform better on this type of rhythm detection task (Figure 2B).

Taking into account the important difference in foil grammars between our experiments and those reported in Shirley (2014), we hypothesize that when given a complex grammar as foil that is not part of the Fibonacci grammatical space, participants would be able to draw upon rhythmic detection abilities to accurately accept grammatical and reject ungrammatical sequences. Success of some individuals on our potentially more difficult task (as compared to Shirley's) already points in this direction. Success in learning Fibonacci grammars using percussion sounds would add support to the claim that rhythm detection is being used to solve this type of Artificial Grammar Learning task, as well as the type of task using speech sounds in Shirley (2014). Future work will address these outstanding issues.

## Overview of the data files and their formats

The raw data files are available at the figshare repository: https://figshare.com/s/83987b4a52906c87e115. The raw data is contained in the file “alldata.csv,” which can be read by any text editor or Microsoft Excel. This file was obtained by merging all output files from individual participants (collected between Dec 5th, 2014 and Feb 26th, 2016), and adding additional information from questionnaire (e.g., musical training). Python scripts used for the analyses are available from the authors on request.

Variable names and coding (values in brackets)

- Experiment_number: Experiment with limited (1) or detailed (2) instructions.
- Condition_id: Participant was tested with Mirror (0) or Swap (1) stimuli.
- Condition_name: alphanumeric string XY indicating the testing condition X and the experiment number Y.
- Participant: anonymized identifier for each participant (1,2,…,40).
- Trial_number: number of trial in order of presentation (1,2,…,36).
- Stimulus_type: test item was a string generated using an L-grammar (0) or a Swap/Mirror string (1).
- Response: Participant judged test stimulus to have the same (1) or a different (0) rhythm as those in the exposure.
- Correctness: participant chose the correct (1) or incorrect (0) response.
- Correctness_by_category: correct acceptance (1) or wrong rejection (2) of a string generated using an L-grammar; correct rejection (3), or wrong acceptance (4) of a string generated by swapping or mirroring elements (depending on the experimental group).
- Goodness: Whether a participant was very sure the sequence was correct (5) or very sure the sequence was incorrect (1) (1,…,5).
- RT: reaction time in seconds.
- Age: Years of age.
- Sex: Female (0) or male (1).
- Musical_Training: Participant had some (1) or no (0) musical training.

## Author contributions

All authors conceived the experiments, designed the stimuli, and edited the manuscript. AG performed the experiments. AR analyzed the data. AG and AR wrote the manuscript.

### Conflict of interest statement

The authors declare that the research was conducted in the absence of any commercial or financial relationships that could be construed as a potential conflict of interest.

## Footnotes

^1^See Martins et al. (2014) for a musical recursion experiment in the melodic domain.
